# The Long Fox Memorial Lecture: Some Considerations on the Cells and Liquids of the Body and the Oxygen-Content of These

**Published:** 1927

**Authors:** George A. Buckmaster

**Affiliations:** Henry Overton Wills Professor of Physiology in the University of Bristol


### The Bristol Medico-Chirurgical Journal

*"Scire est nescire, nisi id me*
*Scire alius sciret."*

AUTUMN, 1927.

### THE LONG FOX MEMORIAL LECTURE:

DELIVERED IN THE UNIVERSITY OF BRISTOL AT THE MEETING OF THE MEDICO-CHIRURGICAL SOCIETY ON NOVEMBER 30TH, 1926.

THOMAS LOVEDAY, M.A., *Vice-Chancellor of the University of Bristol, in the Chair.*

BY

GEORGE A. BUCKMASTER, M.A., M.D. Oxon.
*Henry Overton Wills Professor of Physiology in the University of Bristol.*

ON

### SOME CONSIDERATIONS ON THE CELLS AND LIQUIDS OF THE BODY AND THE OXYGEN-CONTENT OF THESE.

WE have met this evening in all friendliness of thought to carry out the wishes of those who founded a lecture to perpetuate, in this City of Bristol, where he was born and lived, the memory of Edward Long Fox. If the profession of medicine is an exacting one, if

N

those who follow this are compelled, as is the case, to remain students all their lives and reflect upon and regret the narrow limitations of their knowledge, we still possess some compensations. I can think of few which are more sufficing than for a man to have lived through a long life of ceaseless activity and so endeared himself to others, that, year by year, we are enabled to call to memory, if only for an hour, the work and personality of Long Fox.

I must thank those who have invited me to give this memorial lecture. The date of the first is 1904, and this will be the fifteenth occasion on which it has been delivered. Many years ago I had the pleasure of meeting him, was familiar with his writings, and in particular can call to mind his papers on the sympathetic nervous system. It is not necessary here to dwell upon his contributions to medicine, but the singleness of purpose with which he followed his profession is well known to some in this room, and for us all it remains a pleasant memory.

As one of his colleagues has remarked: "He bore ill-will to no one, and was ever ready to show acts of kindness to others, even to those who scarcely deserved it." Is not this almost as great a tribute as it is possible for one man to pay another? Do we not learn how each one of us in different degrees profoundly affects the lives of others? I have ventured to abstract a portion of his address given when President of the British Medical Association which met in Bristol in 1894. From this, better than from any remarks of my own, it will be possible to understand why Long Fox held the position which was conceded to him both as a man and a physician by his fellows.

"As a profession," he said, "we are above party, our highest aspirations tend to the formation of a pure commonwealth. The poor, the sick, the criminal are our daily study, primarily for the relief of the individual. But we have nobler and further reaching aims; that poverty may be mitigated by more healthy surroundings, that sickness may be diminished by the education of the nation in the simple laws of health and in the distribution of knowledge, so that habits of temperance in all things may be increased and by a knowledge from an early age of the common facts of physiology, so that victims of a debased heredity may diminish in our land. Poverty, disease and crime collectively or separately are the subjects of our investigation as a profession, and it should be our aim to remove these."

I propose in this lecture, with your permission, to depart somewhat from the immediate subject-matter of this address, since without extended laboratory experience—I mean complete familiarity with the use of instruments for research—my own special work, which I was given to understand might be of interest to others, is difficult to convey in words without digressions which it would be unwise to pursue.

Years ago, indeed in 1906, when some work on the coagulation-time of normal and pathological blood was undertaken, I devised a method for the study of the coagulation process by using films of blood, which, when held vertically in platinum rings allowed the corpuscles to subside under the influence of gravity and left the plasma free to coagulate at the temperature of the body without any contact beyond the immediate edge of the ring. It was soon noticed that the rate of subsidence was inconstant, indeed showed marked variations. At that time, although I was aware how much attention this phenomenon had received from physicians in the last two or three hundred years, the observation did not arouse my interest or particularly occupy my attention. But the whole question of the suspension stability of the blood has been recently studied by Robin Fåhræus of Stockholm, whose papers, published in 1921, occupy about one-third of Volume LV. of the *Acta Medica Scandinavica.* To an audience which includes many who are actively engaged in medicine, I have thought that a short account of the methods and results of his work might be of interest, since little or none of this is to be found in the manuals or text-books of physiology.

During the years of the war the undoubted value of blood transfusion became of outstanding interest in treatment for hemorrhage. The demonstration by Janski in 1907, together with the work of Moss in America, acquired a direct practical interest, since the frequent dangers, and even fatal consequences, of blood transfusion were for the first time proved to lie in the agglutination or clustering together of red corpuscles and subsequent hæmolysis which occurred in the blood corpuscles of the patient owing to peculiar features in the blood plasma of the donor. Who, by any effort of reason or by any other method than an experimental one, could have discovered that human beings fall into at least four blood groups ? If you will excuse a personal detail I myself am in Group II. These are capable of recognition by no physical or chemical reactions, but only by experiments which are of the nature of biological tests, for strangely enough there are processes in the living body which alone are significant of the presence of agents, the nature and identity of which we are absolutely ignorant. No enzyme, no agglutinin, no hæmolysin has ever yet been seen. It is only by their effects that the existence of these may be affirmed. There is indeed a world of phenomena in physiology as in physics which is beyond our actual recognition in so far as this is dependent on our senses.

Not less interesting is the more recent knowledge which has been gained on the suspension stability of the blood. The treatment of disease even from the earliest times has been related to general pathological ideas. Such views as were held dominated treatment then, just as at the present day. Rational medicine must always be linked up with physiological and pathological knowledge. The two sciences are identical and pursued by identical methods. It is no more possible to have one science for normal and another for abnormal processes of an organism, than it is to divide the study of meteorology into one science for good weather, another for bad weather. All those whose lives are largely passed in laboratory work, whether as chemists, physicists or physiologists, are unwilling to be influenced by opinions, views or ideas, no matter how speciously these are advanced, unless such views are the direct inferences from experiments.

Moreover, the way in which any science grows and develops must always possess an interest, and particularly for those who have to learn how slowly the growth of knowledge may take place. It is well known that from the time of Hippocrates to the middle of the last century both the treatment of disease and the current pathology on which this treatment were based rested to a large extent on the phenomena presented when blood was withdrawn from a patient as a diagnostic or therapeutic measure. Apart from the fact that it clotted, and the rate at which this occurred, the production of a buffy coat was observed in many diseases. This might form a third or more of the clot and appeared to be a dirty white substance, variable in amount, which formed a superficial layer above the rest of the coagulated mass which had clotted like normal blood. This phenomenon dominated the pathology of the day. It is an open question whether, apart from the facts established by Harvey of the circulation of the blood, anything in medical knowledge equalled the importance assigned to the formation of a buffy coat or *crusta sanguinis.* The blood was regarded by all physicians, even as late as Sydenham, to be the seat of an inflammatory process.

Though it was probably little more than a fortunate guess, Theophrastus von Hohenheim, known to his contemporaries as Paracelsus and regarded even from their point of view as medically uneducated—such opinions one may believe are not confined to any particular century—possessed a peculiar kind of intuition which caused him to interpret phenomena in such a way that we must realise he was hundreds of years ahead of his time, for Paracelsus (1538) considered abnormal features of the blood as the result and not the cause of disease. Paracelsus, as you may remember, has been spoken of as divine by Anatole France. Erasmus consulted him for stone. He was the subject of a dramatic poem by Browning. About a hundred years later William Hewson introduced the idea that blood in disease might thicken like size in water. "Sizy blood" became a familiar term in medical literature, as may be seen in the works of John Hunter and Scudamore. It was recognised that sizy blood clotted slowly; blood was therefore drawn as a diagnostic method, and even so late as 1843 Wharton Jones pointed out that it was not necessary to bleed a patient to distinguish between healthy and buffy blood, but that a single drop pressed gently between two glass plates and allowed to settle sufficed to detect the difference. What is the explanation that the whole subject of the buffy coat is to-day little more than a historical curiosity? The reasons for this are partly general, partly special. The middle of the last century was a period of thorough and radical reform both in physiology and pathology, and therefore in the science of medicine. The cellular pathology created by Virchow and his contemporaries brought about in the course of a few decades a complete change in our comprehension of disease. We no longer spoke of inflammation of the blood, nor did we regard it, as for example did John Hunter, as possessed of special vital powers.

When it became obvious that life was bound up with the elementary units of the organism, that is, with the cells of which the body is constructed, even as a cathedral, no matter how magnificent it may be, or what lure it has for lovers of architecture, is built up of separate stones, it followed as a natural consequence that the ultimate causes both of the normal and abnormal performances of the body must be sought for in the cell-changes of the organism. The physiological and pathological importance given to the blood was now transferred to the great cell complexes, in fact to the organs of the body. The reaction against the theories bound up with humoral pathology which had existed for some 2,500 years was so violent that even the facts on which it was founded were attacked. But the facts were correct, it was not the observations which were at fault but the deductions drawn from them. It may be doubted whether Sydenham or Radcliffe, or Trousseau or Graves, to mention a few who have followed the science and practice of medicine, were not the equals of any later clinical physicians.

The remarkable phenomenon of the buffy coat and the changes in its aspect were after a short time forgotten, owing to the almost complete disappearance of blood-letting as a therapeutic measure, and it is chiefly due to the researches of Fåhræus that a renewed interest has been awakened in the question of how a buffy coat arises in shed blood, for this, as we shall see, is due to an abnormal suspension stability of the blood.

Blood, as is well known, is so constituted that physically it is a coarse reversible suspension of corpuscles in plasma. When the suspended bodies have a higher specific gravity than the suspension medium, these will fall with a velocity which can be calculated when the suspension remains undisturbed, as was shown by Stokes in 1850. For example, small gold particles 1 $\mu$ radius will fall 2·4 mm. per minute, that is, the suspension will clear to the extent of 14 cm. in one hour, but with particles which are only one-hundredth the size these will subside only 10 mm. in a month.

In the method employed by Fåhræus blood was citrated and allowed to stand in tubes 17 cm. in length and 9 mm. wide. Great variations were observed in subsiding speed. At first this is slow, but acquires a steady definite rate in about twenty minutes. Obviously the better the stability the slower the rate of settling, and as a measure of this Fåhræus observes the length in millimetres of the layer which is clear of corpuscles at the end of one hour. The value of this indicates the suspension stability, and the higher the values the less is the stability of the blood suspension. The suspension stability is inversely proportional to the sinking rate.

The stability reaction has been investigated in about 400 cases of different physiological conditions with the following results. The higher values indicate diminished stability. The following are average results in physiological conditions :—

| | | |
|---|---|---|
| 7 children (the blood was obtained from the umbilical cord at birth) .. .. | 0·5 | mm. per hour. |
| 84 men .. .. .. .. | 3·3 | ,, ,, |
| 61 women .. .. .. | 7·4 | ,, ,, |
| 48 (parturient, pregnant) women | 44·9 | ,, ,, |

From these figures it may be concluded that human beings can be classified into groups which are regulated by age, sex and physiological conditions. The conditions in pregnancy are such that the speed of sedimentation rapidly increases month by month.

| Suspension Stability in mm. | | Month of Gestation. |
|---|---|---|
| 6 | .. .. .. .. | 1 |
| 17·4 | .. .. .. .. | 2 |
| 23·8 | .. .. .. .. | 3 |
| 20·7 | .. .. .. .. | 4 |
| 29·2 | .. .. .. .. | 5 |
| 33·3 | .. .. .. .. | 6 |
| 40·7 | .. .. .. .. | 7 |
| 47 | .. .. .. .. | 8 |

Individual variations of course exist, but evidently the well-known fact that in pregnancy the blood is profoundly changed remains confirmed. Further, the reduced suspension stability attains its maximum at the close of pregnancy and remains for at least two months of the puerperium at an average of 41 mm. for the first and 19·5 mm. per hour for the second month.

Fåhræus regards rates greater than 9 mm. for men and 12 mm. for women as pathological, and his extended researches, which have been to a considerable extent confirmed by Linzenmeyer and other observers, also show without exception that the suspension stability of the blood is reduced in almost all diseases, and this reduction is considerable. Time allows only a few additional details to be mentioned. The number of cases investigated is shown in brackets after the name of the disease.

### INFECTIOUS DISEASES.

Septicæmia (2 cases): 95 mm., 105 mm. per hour.

Typhoid fever: second week, 23 mm. per hour.
fourth week, 49 mm. per hour.
eighth week, 75 mm. per hour.

Pulmonary tuberculosis (3 cases): 40, 60, 85 mm. per hour.

Croupous pneumonia (4 cases): 50, 53, 68, 80 mm. per hour.

Evidently from these experiences of the stability reaction the reduction is almost pathognomonic. Westergren, among others, has employed the methods suggested by Fåhræus and confirmed in a comprehensive research that not only is the sinking velocity abnormally high in pulmonary tuberculosis, but that the extent of this change in the blood runs parallel to the degree of severity of the disease. After injections of tuberculin the sinking velocity also rises definitely after each injection. After uncomplicated fractures or simple operations the suspension stability shows diminished values of 22 mm., 25 mm. and 29 mm. per hour.

When the cause of variations in the speed of sedimentation was investigated, Fåhræus, as the result of careful experiments, came to the conclusion that changes in the viscosity of the plasma, of the sizes of corpuscles, of the specific gravity of the liquids were practically negligible in the process of sedimentation. The phenomenon is due to agglutination of corpuscles. It is known that red corpuscles easily run together into the well-known rouleaux formation or agglutinate into masses. Both these states can easily be augmented or diminished. Simple laboratory experiments conclusively prove this fact, and in disease exactly similar states may occur.

It is possible to calculate that while a single red corpuscle or red disc will fall through a large volume of plasma at a rate of 0·2 mm. in an hour, in the blood, where the volume of corpuscles to plasma is as 1 : 1·4, sedimentation would take place at about one-tenth of this rate. With a sedimentation rate of 1 mm. an agglutinated clump of eleven corpuscles will fall with a velocity of 1 mm. per hour, and a clump of 58,000 at a rate which gives a suspension stability of 75 mm. and a sinking velocity of 300 mm. per hour.

Fåhræus is of opinion that the cause or causes of agglutination are not due to any specific substance in blood plasma. It is possible or even probable that specific agglutinins exist in pathological states, but in normal individuals the agglutination process is identical with the rouleaux formation of red corpuscles and is indeed only an exaggeration of this phenomenon which is one that always occurs in shed blood.

The degree to which this takes place is chiefly dependent upon the properties of the plasma. Of the constituents the colloidal complexes are the most potent, the proteins fibrinogen and globulin; of these the former, though existing in human blood to only the extent of 0·56 per cent., is particularly efficient as an agglutinating agent. This is shown by the fact that the suspension stability of defibrinated blood is much greater than blood which has been hindered from coagulating by treatment with sodium citrate. That the speed of sedimentation is determined by some constituent protein in plasma, and not by any features of the suspended corpuscles, may be inferred from experiments where the addition of an emulsive colloid such as gelatin to blood accelerates the sinking rate, whereas if plasma be diluted with a solution of inorganic salts, as when Ringer's fluid is added, the rate of subsiding is reduced and the suspension stability augmented.

Some experiments made by Fåhræus, in which the corpuscles and citrated plasma of two blood tests which showed considerable differences in stability were simply exchanged, possess considerable interest.

EXPERIMENT I.

| Suspension Medium. | Rate of Sedimentation. Male Corpuscles. | Corpuscles of Pregnancy |
|---|---|---|
| Plasma (male) .. | 4 | 10 |
| Plasma (pregnancy) | 55 | 62 |

EXPERIMENT II.

| Suspension Medium. | Rate of Sedimentation. Male Corpuscles. | Corpuscles of Pregnancy. |
|---|---|---|
| Plasma (male) .. | 8 | 9 |
| Plasma (pregnancy) | 54 | 67 |

EXPERIMENT III.

| | | |
|---|---|---|
| Plasma (male) .. | 16 | 21 |
| Plasma (pregnancy) | 45 | 50 |

From these figures the conclusion may be drawn that the different degrees of agglutination of the red corpuscles and the variations in sinking speeds are chiefly, if not entirely, dependent on the properties of the plasma and but to a small extent on the properties of the corpuscles.

It is clear that changes in the suspension stability of the blood must be accepted as an important physiological and pathological fact. Is it justifiable to apply the information gained by laboratory experiments when the flow of blood within the vessels of living animals is considered ? This subject, in view of the frequent occurrence of post-operative thrombosis, well deserves extended research, and moreover the subject gains in interest should it be found that we are able to influence this stability during life. This reduced suspension stability is almost certainly associated with an increased tendency to coagulate.

It is known from Lombard's original observations some years ago that the cutaneous capillaries can be microscopically studied in reflected light. When the capillary flow of a healthy man or woman is contrasted with that of a pregnant woman a marked difference may be observed. A still more striking contrast may be seen in the blood flow of a pneumonic patient. The minute blood stream appears coarser, more granulated, the granules are not equally distributed and sometimes the stream appears as if fractured in pieces. This appearance is produced by the corpuscles being agglutinated in the streaming blood. This liquid possesses, in fact, during life a diminished suspension stability.

The appearance of hemagglutination in living blood has also been studied by Ploman at the suggestion of Fåhræus. Here the streaming of blood in the retinal vessels was observed with the ophthalmoscope. It is only possible here to remark that under certain conditions of experimental pressure on the eyeball Ploman succeeded in comparing the stability reaction with the ophthalmoscopic picture, where countless transitions varying from a finely-granulated sandiness in the retinal vessels to a complete resolution of the blood column could be observed. It may therefore be considered as certain that the reduced suspension stability of the blood may be demonstrated during life, and that this is evidenced by increased agglutination of the red corpuscles.

When we reflect upon the unexpected dangers which may be associated with or dependent upon an augmented rate of sedimentation in consequence of stagnation of the blood or of thrombosis within the blood vessels, we realise that such studies as these by Fåhræus, even if a completely final answer is not given to the problem, are of significant interest. Thrombosis in pregnant women, in influenza, in pneumonia, and in states which may occur after operations is by no means uncommon. Death may occur almost instantaneously, or within a few minutes without warning. Research which has for its object a direct application to medicine is among the most fascinating of pursuits. May it not also be regarded as well worth the time and labour which must be freely given.

With your permission I would like in the briefest possible way to refer to some work which is still in progress in this physiological laboratory, the equipment of which in apparatus and material is second to none in this country. Cynics have spoken of the present time as one of large laboratories and small men, when contrasted with the small inadequate rooms in which such men as Pasteur, Claude Bernard and Paul Ehrlich carried out their early work—the age of great men and small laboratories. As one of the Henry Overton Wills professors, may I here simply acknowledge a debt due for all time to him and also to his sons — individually and collectively — *benefactores nostri.*

Since the discovery of oxygen by Priestley in 1774, the nature of the process of combustion by Lavoisier, in other words oxidations in the inorganic and organic world, and the subsequent introduction by John Dalton, who through a long life earned his bread by teaching at half a crown an hour, of Lavoisier's idea of weight into chemical science, the progress of physiology has been closely dependent upon advances in chemical and physical sciences, and this view includes pathology, though in this subject, for special reasons, the study of parasitic protozoa and protophyta has acquired exceptional prominence.

The processes of life in all living things are dependent upon a continuous supply of oxygen. Man cannot store this in his body. He possesses a store just sufficient to last for four minutes. This would appear a somewhat alarming state of things when we realise that chemical energy is the source of all those transformations of energy which are capable of being effected by living things, and which exhibitions of energy are indeed the outward and visible signs of life.

Any researches which are concerned with the behaviour of oxygen therefore attempt an intimate study of the fundamental physico-chemical nature of oxidation processes as these are manifested during life. Regarding the respiration of pure oxygen, we may remember that some thirty years ago Rudyard Kipling, when seriously ill, respired this gas for many days, and here I would wish to recall the original observation of Priestley, for he had the curiosity to breathe the gas himself, which he had discovered ;

"The feeling of it to my lungs was not sensibly different from that of common air ; but I felt that my breast felt peculiarly light and easy for some time afterwards. Who can tell but that in time this pure gas may become a fashionable article in luxury ? Hitherto only two mice and myself have had the privilege of breathing it. . . . But, perhaps we might infer that though pure air might be very useful as a *medicine* it might not be so proper for us in the usual state of the body, for, as a candle burns out much faster in pure than in common air, so we might *live out too fast.* A moralist, at least, may say, that the air which nature has provided for us is as good as we deserve."

The gases of the atmosphere are, as is well known, found in the cells and liquids of the animal body. Though the presence of gases in the blood was first shown by Boyle in 1636, even as late as 1830 their existence in this liquid was denied, even by so distinguished a physiologist as Johannes Müller. Davy, some of whose researches were carried out in the Hotwells district of Bristol, had, however, pumped out the gases of blood. Not to physiologists, but to physicists, are we indebted for a complete demonstration of the quantity and nature of the blood gases. In 1837 this was first given by Wilhelm Magnus, of Berlin, repeated by Lothar Meyer, and, with the device of a special vacuum pump, the first physiological blood gas pump was introduced into experimental work. This was, as indeed were most of the succeeding forms of apparatus, difficult to work, and unavoidable errors had to be discounted, and in particular the values obtained required considerable adjustment. Most, if not all, of the pumps allowed a leak of air. J. A. Gardner and myself, when investigating the distribution of anæsthetic vapours in the body, had to carry out a long series of duplicate gas analyses, and devised a form of tapless leak-free pump which has been used during the last twenty years. It is because of familiarity with its working and the belief in its accuracy that I venture briefly to state a few of the results of experiments made in this laboratory. Dr. Hickman and myself have carried these out, more than a hundred in number.

The condition of oxygen in the blood is well understood at the present time. Its quantity, its pressure, the physico - chemical relations of the gas and liquid are firmly established among a somewhat conflicting mass of physiological work. But the condition of oxygen in liquids of the body other than the blood or of the living cells themselves is not so definitely known. For the latter our knowledge is practically confined to one cell-mass of the body, to the voluntary muscles. From these or from the liquids bathing the cells only carbon dioxide can be obtained. When we think of living cells, it becomes evident that it is not the mass of the solid substances in these which is the important thing, but the whole colloid complex which can only exist with water. In the case of nerve cells, 9,000,000,000 of which are considered to form the cerebral cortex, there is only 14·18 per cent. of solids, the rest is water holding among countless other molecules, molecules of gas. What is the quantity and tension of the gases held by cells ? An answer to this question cannot be given with any degree of scientific accuracy. But it is a question that must be faced. According to some observers, in the cells of the salivary gland the pressure of oxygen is comparatively high, in the muscles it is low. In all living cells the oxygen pressure is clearly the limiting factor for the oxidation-processes, but, as August Krogh has indicated, a great deal of exceptionally difficult work will have to be done before we can say anything quantitatively about the relation between oxygen tension and the rate of oxidations in the warm-blooded organism.

Reflecting on this question, it seemed to us that those liquids of the body which are in contact with living cells might conceivably be in such an equilibrium, that indirectly some information might be gained, if experiments were carried out on the gas-pressures of those liquids. We have therefore examined those liquids which are formed by the liver and kidneys and which are stored in contact with a many-layered cell-mass lining the hollow reservoirs in which these liquids are stored.

Without coming in contact with the air, large quantities, 174 c.c., of these liquids which were warm and fresh were introduced into a tapless blood pump, which was of high efficiency, the last traces of air being removed with cocoa-nut charcoal kept at a low temperature. The results of some experiments are indicated in the following series :—

| | c.c. | Total gas evolved in c.c. | c.c. of gas in 100 c.c. N.T.P. $CO_2$ | $O_2$ | $N_2$ |
|---|---|---|---|---|---|
| Bile. | 85·00 | 12·85 | 12·67 | 0·22 | 1·00 |
| | 78·50 | 22·73 | 24·91 | 0·89 | 1·95 |
| | 174·08 | 20·43 | 9·72 | 0·59 | 0·64 |
| | 174·08 | 22·93 | 10·94 | 0·09 | 1·10 |
| | 174·08 | 24·08 | 11·21 | 0·19 | 1·03 |
| Urine. | | | | | |
| | 174·08 | 13·93 | 6·44 | 0·24 | 0·90 |
| | 174·08 | 14·35 | 6·08 | 0·29 | 0·84 |
| | 174·08 | 17·25 | 7·63 | 0·28 | 1·42 |
| | 174·08 | 29·40 | 13·96 | 0·25 | 1·52 |
| | 103·15 | 5·70 | 3·77 | 0·63 | 0·84 |
| | 174·08 | 12·95 | 5·12 | 0·33 | 1·52 |
| | 174·08 | 10·95 | 4·28 | 0·46 | 1·13 |
| | 174·08 | 12·70 | 5·39 | 0·23 | 1·03 |
| | 109·00 | 6·45 | 4·17 | 0·39 | 1·04 |
| | 93·00 | 5·55 | 4·26 | 0·28 | 1·26 |

Earlier observers have also recorded a few values for the oxygen-pressure of milk and saliva. Since no liquid of the body, except blood, can hold oxygen gas in any form but that of simple solution, it is easy from known co-efficients of solubility to estimate the tension of this gas. The quantity found by Pflüger was 0·66 per cent. of oxygen in saliva, and this would correspond with an oxygen pressure of over 200 mm. of mercury. Our own figures we consider give an average value of 0·33 per cent., and this would indicate an oxygen pressure which is more than 100 mm. of mercury. It is conceivable that the oxygen pressure within the cells lining the bladder is in equilibrium with that of the liquids, and should this ultimately be found to be the case, then a minute amount of oxygen within the living cells might in virtue of its high potential be capable of bringing about, at the temperature of the body, that mysterious process of oxidation which is the characteristic feature of cell activity.

Michael Foster, to whom English physiology owes so much, and of whose almost boyish spirits I have so clear a memory, has written at length on what is to-day a physiological myth — the existence of an intra-molecular store of oxygen. Although this view is no longer supported by experimental work, his concluding words must and will do well, better than anything I can pretend to say, to show how little is known, how much to know:

"We cannot as yet trace out the steps taken by the oxygen from the moment it slips into its intra-molecular position to the moment when it issues united with carbon as carbonic acid. The whole mystery of life lies hidden in the story of that progress, and for the present we must be content with simply knowing the beginning and the end."

